# Transformational leadership and project success: The mediating role of trust and job satisfaction

**DOI:** 10.3389/fpsyg.2022.954052

**Published:** 2022-09-15

**Authors:** Muhammad Zeeshan Fareed, Qin Su, Mubarak Almutairi, Kashif Munir, Mian Muhammad Sadiq Fareed

**Affiliations:** ^1^School of Management, Xi’an Jiaotong University, Xi’an, China; ^2^State Key Laboratory for Manufacturing Systems Engineering, Xi’an, China; ^3^The Key Laboratory of the Ministry of Education for Process Control & Efficiency Engineering, Xi’an, China; ^4^College of Computer Science and Engineering, University of Hafr Al Batin, Hafar Al Batin, Saudi Arabia; ^5^Department of Computer Science, Khwaja Fareed University of Engineering and Information Technology, Rahim Yar Khan, Pakistan

**Keywords:** project success, transformational leadership, trust, job satisfaction, public sector

## Abstract

Transformational leadership (TFL) impacts on project and organizational success are well established. However, many underlying factors that make TFL effective are still missing. Therefore, we formulated hypotheses and tested the mediating role of trust (TS) and job satisfaction (JS) in linking TFL to project success (PS). A time-lagged methodology was used to collect quantitative data using a structured questionnaire from 326 project manager-team member dyads working in Pakistan’s public sector. Our results showed that TS, JS, and TFL significantly impacted project success. Moreover, we found that TS and JS mediate the relationship between TFL and PS. These findings highlight the importance of trust and job satisfaction as mechanisms that translate TFL into the success of projects for organizations.

## Introduction

Transformational leadership (TFL), possibly the most studied leadership theory to date, is closely associated with anticipated outcomes for people (Braun et al., 2013). Researchers worldwide have delivered evidence of the positive influence of TFL on project success (PS), work outcomes, and organizational success. Yet, a proper comprehension of leadership effectiveness also needs an understanding of the psychological processes that mediate the impact of a leader’s behavior on followers’ reactions to them (Zhu et al., 2013; Nübold et al., 2015). While the findings of scholars vary in some aspects, all of them identify job satisfaction (JS) of followers and trust (TS) creation as vital components of the transformational leader-follower relationship. Scholars have revealed that trust fully mediates the influence of TFL on job performance (Jung and Avolio, 2000). Yet, TFL’s indirect effects (through the creation of trust and job satisfaction) on public project success have never been investigated before.

Social exchange theory (SET) (Blau, 1965) has been used extensively to elucidate the effect of TFL on subordinates’ job performance (Dirks and Ferrin, 2002). SET states that when a leader treats his employees well, in return, they make more significant efforts in the organization’s interest (Organ, 1988). To evaluate the social exchange quality between leader and follower, trust in the leader has been extensively used by scholars (Lavelle et al., 2007). The amount to which employees are willing to subordinate themselves to the leader’s actions determines how they are treated by their leader (Zhu et al., 2013). Transformational leaders must build superior trust levels in employees when they show encouragement, support, respect, and concern for their subordinates (Jung and Avolio, 2000; Dirks and Ferrin, 2002). Employees generally put more effort into completing job tasks timely and are possibly engrossed in behaviors that benefit their organization, even when their specific role is not to engross in those behaviors with excellent trust levels in the leader (Organ et al., 2006; Burke et al., 2007). For instance, a previous study (Organ et al., 2006) has revealed that employees in a trusting relationship exchange in the shape of performance, superior work attitudes, and organizational citizenship behavior. Dirks and Ferrin’s (2002) meta-analytic work revealed similar findings for a significant relationship between leader trust and employees’ job performance.

In leadership research, the dearth of context specificity has been extensively criticized (Braun et al., 2013). Consistently, this research aims to extend TFL research and its implications to a leadership context that has not been previously studied, that is, the public sector of developing countries. Our focus is on public projects, as they have a substantial direct impact on our society. In low-income and high-income countries, managements devote large amounts of funds to public projects each year (Flyvbjerg, 2014). In recent years, a higher rate of project failure has been noted regardless of research on technical features, for example, risk, cost, portfolio management, and timing (Imam and Zaheer, 2021); this percentage is higher in developing countries than in developed countries (Gazder and Khan, 2018). Project managers (PMGs) approach the project in their own way as they are the leading driving force behind the project, and the project team’s work largely depends on their leadership style (Gruden and Stare, 2018). The project’s failure or success is much dependent on its leader (Drouin et al., 2018; Raziq et al., 2018).

Transformational leaders’ impact on public management is widely established (Trottier et al., 2008; Vogel and Masal, 2015); however, one of the critical success factors (CSFs) is the operationalization of TFL in projects (Raziq et al., 2018). TFL is a leadership style that may be appropriate for the project context (Yang et al., 2011; Kissi et al., 2013). Therefore, this research has two research questions. First, several scholars have studied the direct influence of TFL on PS; however, does TFL indirectly (e.g., trust and job satisfaction) influence PS? Second, do trust and job satisfaction affect employees and organization performance, and do trust and job satisfaction influence project success?

In particular, consistent with the underlying principles of SET, we claim that trust and job satisfaction building may mediate the relationship between TFL and project outcomes. We believe that transformational PMGs may build more trust and increase the job satisfaction of their team members (TMs). We then argue that upper trust levels and job satisfaction, in turn, lead to higher levels of project success in terms of effective problem-solving, top management support, high-quality communication, and task clarity (Mazur et al., 2014). We also note that Gilstrap and Collins (2012) found TFL to be an antecedent for trust and job satisfaction. In our research, we augment these findings by examining variables in public project studies. In this respect, Judge et al. (2001), Pheng and Chuan (2006), and Thompson (2011) found a positive relationship between job satisfaction, trust, and project success. The literature review indicates that no study has examined the mediating relationships associated with these variables in the context of public projects. We believe that our research adds to practice and theory in three ways. First, we developed and empirically tested a model of the effect of TFL on a sample of TMs and managers working on public projects. Second, we explored the underlying mechanisms by which transformational PMGs can contribute to project success. Third, we contribute to the growing literature on the affective, behavioral, and attitudinal effects of TFL in public sector projects.

## Hypothesis development

### Project success

Project success is determined by the performance of its various dimensions, for example, time, budget, and quality of final results, amongst others (Aubry, 2015). To date, there is no concord in the PM literature concerning the appropriate criteria for measuring PS (Khosravi et al., 2020). “The traditional definition of project success, which revolves around time, cost and quality, proved to be inadequate” (Pheng and Chuan, 2006, p. 25). According to Besteiro et al. (2015), describing PS is not easy and is influenced by the stakeholders’ perception, the project type, the time perception, and the organization. Davis (2016) has produced a group of three new constructs of project success that comprise the client or customers’ concerns, stakeholders’ benefits, and the standard dimensions of quality, cost, and time. Albert et al. (2017) explored the topic of PS in various areas in the literature, and the authors defined that the success criteria were performance, time, and cost, and they also included economic success and quality.

The methods of measuring and achieving project success have evolved over the years. First, the literature used iron triangles for project evaluation, then a CSF list was created, and the first success framework was presented (Tam et al., 2020). Some researchers tried to measure PS through team performance (Tabassi et al., 2017) or the project management (PM) method (Carvalho et al., 2015). Concluding all, a huge body of literature has catered to many CSFs for PM in an organizational context. Several researchers emphasized founding a set of success factors, while others concentrated on developing a relationship between CSFs and PS (Jitpaiboon et al., 2019). This study has used Musawir et al.’s (2017) scale to measure PS, as this scale has all three constructs of project investment success (PIS), project ownership success (POS), and project management success (PMS). Moreover, this scale has been validated and proved to be reliable.

### Transformational leadership and project success

The most popular leadership concept since the 1980s has been TFL theory, which has unswervingly shown a substantial influence on multiple organizational standards and outcomes, for example, commitment, JS, performance, and TS (Hoch et al., 2018). In the last three decades, most leadership research has focused more on TFL, which offers resilient support for employee wellbeing, organizational climate, culture, project team, and organizational performance (Zaman, 2020). Transformational leaders provide a full description of the project’s future and promote stakeholder rendezvous that eventually lead to PS (Kissi et al., 2013; Maqbool et al., 2017). TFL also promotes a high level of cohesion, extraordinary engagement, and coordination within the project team to ensure PS (Kissi et al., 2013; Aga et al., 2016; Raziq et al., 2018). TFL positively impacts employees, instills ethics and high values, and stimulates the project team’s energies and emotions to complete the organization and the project’s objectives (Pieterse et al., 2010).

Robbins and Judge (2013) believed that TFL is one of the most significant theories of this century. Burns (1978) intellectualizes TFL has four distinct constructs, specifically idealized influence (II), intellectual stimulation (IS), inspirational motivation (IM), and individualized consideration (IC) (Bass, 1985; Bass and Avolio, 1994). Many studies suggest that the TFL style is better than the transactional leadership (TSL) style in accomplishing the project and organizational objectives (Müller and Turner, 2010; Raziq et al., 2018). Gardner and Stough (2002) and Zareen et al. (2014) studied different leadership types and suggested that TFL is more effective and influential in different settings than laissez-faire and TSL.

Transformational leaders can produce an environment where TMs exert their utmost efforts for PS (Burke et al., 2006). Piccolo et al. (2010) revealed a constructive association between TFL and PS. TFL and its different measures play a leading role in refining team collaboration, influencing TMs and team member performance to achieve anticipated tasks (Hassan et al., 2017). Therefore, the PS rate is higher (Amin et al., 2016).

The PMG’s skills and TFL are positively correlated with project success (Maqbool et al., 2017). The TFL style plays a prominent role in improving the team’s effectiveness, and work inspires the team and makes them follow the leader’s actions, ultimately leading to PS. Under TFL, the team thrives and practices new ideas and creativity that make employees more productive and dedicated to a particular organization, indirectly and directly guaranteeing a particular project’s success (Tabassi et al., 2017). PMGs who have adopted a TFL style tend to be more productive and successful in providing a work environment that promotes employees’ safety, welfare, and wellbeing. We see that a PMG acquires a transformational leader’s traits, can satisfy workers, and meet deadlines on time, as employees trust and respect these managers the most (Boamah et al., 2018). Keeping the literature and context of study in mind, we propose the following hypothesis:

H1: Transformational leadership positively and significantly influences project success.

### Trust and project success

Trust contributes to enriched performance and positive organizational citizenship behavior (Lewicki et al., 2006; Colquitt et al., 2007). Tyler (2003) states that trust impacts performance by activating cooperation or other collaborative processes. Trust teams foster collaborative and cooperative approaches, which help them manage the interdependence between their own areas of expertise (Rezvani et al., 2016). PMG’s trust in the stakeholder improves problem-solving, communication, and organizational support (Diallo and Thuillier, 2005). Trust is linked to project success (Cerić et al., 2021). Trust is often studied in terms of its impact on project success through developing high-performing teams and improving efficiency (Gad and Shane, 2014).

TMs mostly rely on a trusted PMG to take action and achieve desired results (Khosravi et al., 2020). Most would approve that trust in a project’s context is anticipation concerning the behaviors and actions of others (Wu et al., 2017). Trust is also seen as an organizational principle that provides particular benefits to teams that motivate anticipated performance and positive behavioral outcomes (Pinjani and Palvia, 2013). Trust fosters collaboration and communication and mobilizes TMs to contribute value-adding resources (Cheung et al., 2013). Consequently, in a trusted atmosphere, they possibly construct collaborative relationships that motivate superior project performance (Khosravi et al., 2019). Therefore, we propose our second hypothesis:

H2: Team members’ trust in project managers positively and significantly influences project success.

### The mediating role of trust

Trust fosters internal motivation that facilitates cooperation and promotes openness (Agbejule et al., 2021). TS as a variable in the relationship between the manager and the teamwork reciprocated and includes the team member’s trust toward his supervisor and vice versa (Ozyilmaz, 2010). “Trust in the Chinese perception is a social phenomenon that can bring harmony, it is believed to be a good and positive relationship that one should develop and maintain, but too much trust will also bring disaster if the trusted party does not perform” (Lau and Rowlinson, 2011, p. 634). Bass (1985) adopted a TFL theory that builds on earlier work by Burns (1978). The degree of leaders’ transformation was deliberated apropos of the leader’s influence on employees (Barbuto, 2005). Subordinates of transformational leaders feel TS, loyalty, respect, and admiration for leaders and are enthused to engage in additional role behaviors (Katz and Kahn, 1978; Bass, 1985). Transformational leaders have been proven to escalate follower satisfaction with citizenship and trust (Podsakoff et al., 1990). Transformational leaders’ behaviors are generally seen in people who believe in the organization’s purpose and trust (Katz and Kahn, 1978; Bass, 1985). Intrinsic motivation embodies a person and their emotions and includes pleasure, TS, and self-esteem, all of which result from internal effects (Barbuto, 2005). These traits are related to those required for transformational behaviors (Bass, 1985, 1990). A central mechanism of an effective TFL process is the growth of followers’ TS in the leader (Podsakoff et al., 1990; Jung and Avolio, 2000; Kark et al., 2003). TS has typically been used to evaluate the SE quality between leader and follower (Pillai et al., 1999; Schaubroeck et al., 2011).

A leader with TFL offers exemplary effect by serving as a role model and must garner superior TS levels from employees (Jung and Avolio, 2000). A willingness to set team objectives on personal benefits and exhibiting exemplary behavior by the leader would make the emotional bond stronger between follower and leader, leading to upper levels of emotional trust. Furthermore, a leader who exhibits a willingness to immolate individual merit for team objectives keeps uniformity between his actions and spoken words, improves followers’ perceptions of his trustworthiness and integrity, and generates higher TS levels (Avolio and Bass, 1995; Kirkpatrick and Locke, 1996). Similarly, a leader with TFL exhibits IS, and must generate greater trust levels in employees. By encouraging and fostering creativity, a leader empowers their subordinates to participate in the decision-making procedure and allow them to affect decisions that can affect them. This delivers a signal to followers that the leader respects them and is willing to engage in SE (Avolio and Bass, 1995). This reciprocation will make the emotional bonds resilient between the two groups and lead to superior TS levels. Such behavior is expected to build TS because it should improve followers’ perceptions of their leader’s competence, integrity, and trustworthiness. Showing IM on the part of a transformational leader in creating a shared vision that TMs can recognize and confirming that vision is achieved must increase follower confidence (Avolio, 1999). When employees have a clear comprehension of how their individual actions can contribute to its accomplishment and their leader’s vision for the organization, they will be more enthusiastic to engage in the course of SE (Pillai et al., 1999). This must take to greater TS levels. The inspirational leader’s values and a better understanding of followers grow TS, which fortify the emotional bond between them (Lewicki et al., 1997).

Furthermore, suppose a leader is able to accomplish his or her vision. In that case, this must lead to a greater perception among employees that their leader is a trustworthy, capable, and reliable manager who can smoothly achieve organizational objectives, which will generate TS. The offering of IC by the transformational leader must generate higher trust levels in his employees. Since TS results from an employee’s ascription that the leader sincerely cares about employees and works with their best interests in mind, leaders with TFL demonstrate care for followers’ wellbeing, requirements, and job security, which will further strengthen emotional bonds with employees and generate superior TS levels (Jung and Avolio, 2000; Dirks and Ferrin, 2002). Leaders who demonstrate individual deliberation are also more likely to improve followers’ perceptions of the leader’s character in terms of their competence, trustworthiness, and integrity, leading to greater levels of cognitive TS.

Previous studies propose that TS may mediate the relationship between TFL and subordinates’ behaviors, as it exemplifies the procedure that happens when leaders involve in SE with followers (Yang and Mossholder, 2010). TFL behaviors must help leaders cultivate close emotional relationships with their subordinates, leading to greater levels of emotional TS. This will allow subordinates to perceive the actions of their leaders as real, improve their experience in the place of work, and produce positive attitudes toward their work, such as emotional and organizational commitment (Yang et al., 2009). Furthermore, when leaders with TFL show concern and care for their subordinates, the latter must, in return, engage in more role behaviors that their leaders want, for example, by improving JP (Dirks and Ferrin, 2002). Transformational leaders should also enhance team cohesiveness by smoothing interactions among co-workers, making subordinates more relaxed in each other’s presence, and resulting in a larger willingness among them to go above and beyond their role to help each other and your organization on a voluntary basis (i.e., demonstrate superior OCB levels) (Konovsky and Pugh, 1994; Burke et al., 2007; Yang and Mossholder, 2010).

H3: Team members’ trust in project managers has a positive mediating effect on the relationship between transformational leadership and project success.

### Job satisfaction and project success

Job satisfaction is an emotional state that results from an appraisal or evaluation of an individual’s work experiences (Locke, 1970). Job satisfaction is the outcome of two kinds of factors: “intrinsic” and “extrinsic” (Herzberg, 1968). Manager’s leadership is an external factor that substantially impacts a follower’s work attitude. A leader’s positive attitude toward followers increases followers’ attitude toward their leader, work, and the organization. In turn, employees cultivate intrinsic motivation. Good combination of extrinsic and intrinsic motivation results in JS and project or organizational success (Mardanov et al., 2008). Employees who are contented with their jobs show higher job performance (Jones, 2006). Scholars have constantly associated the concept of JS with the success of business operations and performance (Judge et al., 2001; Jones, 2006).

Satisfied employees perform well because of the easily accessible experiences that make them feel more satisfied when they perform work tasks more effectively and underperform when they are less satisfied (Fisher, 2003). Several scholars have revealed that JS is a substantial component in PMGs’ performance and PS (Pheng and Chuan, 2006; Bowling, 2007; Rezvani et al., 2016). Pheng and Chuan (2006) revealed that job satisfaction is one of the ingredients of PMGs’ performance that affects PS, especially in complex projects. JS is a motivation for PMG, which leads to the PS. Rezvani et al. (2016) underlined the impact of PMGs’ trust and JS on project success of complex projects.

H4: Job satisfaction positively and significantly influences project success.

### The mediating role of job satisfaction

Transformational leaders are believed to improve their followers’ JS by making followers feel special (through IC) and by making followers feel called to a superior objective (through superior motivation and influence) (Bass, 1985). Individual thinking makes employees feel esteemed, and their need to comprehend and resolve their personal concerns about integrity is respected (Bass, 1998). As JS is a key performance precursor (Spector, 1997), it is treasured to comprehend what elements contribute to it. Studies have indicated that one such element is employees’ perceptions of their leaders. For instance, perceptions of leader ethicality (Brown et al., 2005), exchange quality (Scandura and Graen, 1984), support (Erdogan and Enders, 2007), and trust (Aryee et al., 2002) are positively related to employee job satisfaction. Transformational leaders display IC and are thus able to identify and respond “to each individual’s abilities, aspirations, and needs” (Walumbwa et al., 2005, p. 238). TFL has been positively associated with JS in several studies (Podsakoff et al., 1990, 1996; Judge and Piccolo, 2004). Leaders with TFL modify their IM and idealized influencing behavior according to the explicit aims and benefits of individual subordinates (Chun et al., 2009) and confirm that each employee can articulate their concerns through mentally stimulating behavior (Liu et al., 2010). These transformational behaviors result in employees being more satisfied with their jobs as they trust that the work they are doing is important to leaders and their leaders value these contributions (Nemanich and Keller, 2007). The fact that TFL is directly related to job satisfaction has already been proven (Podsakoff et al., 1990; Judge and Bono, 2000; Judge and Piccolo, 2004). Therefore, we conclude that the followers’ JS will depend partly on these direct and related individual experiences with their managers. Consequently, we propose our last hypothesis:

H5: Job satisfaction has a positive mediating effect on the relationship between transformational leadership and project success.

**FIGURE 1:**
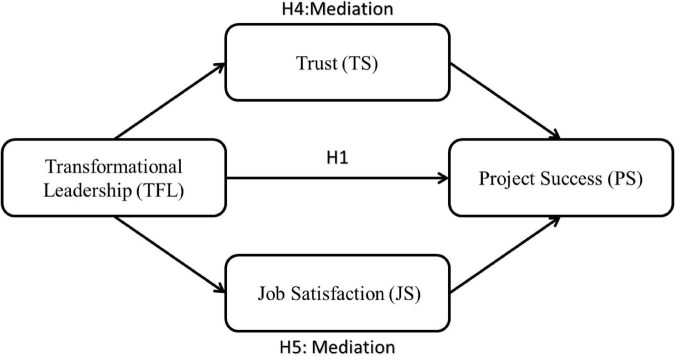
Research model.

The previous discussion leads us to the explanatory paradigm and the hypotheses proposed in Figure 1.

## Research method

To examine the theoretical model, this research used post-positivist philosophy. Post-positivism “assumes that the world is mainly driven by generalizable (natural) laws, but their application and results are often situational dependent. Postpositivist researchers, therefore, identify trends, that is, theories which hold in certain situations, but cannot be generalized” (Biedenbach and Müller, 2011, p. 87). The post-positivist approach suits well with social science research, and it has appeared as the main philosophy of quantitative research in the social sciences (Teddlie and Tashakkori, 2009). To collect quantitative data, a structured questionnaire was used.

### Data collection

We used a time-lagged approach to collect data from TMs and PMGs working on various Pakistan public projects. Contact details of project directors employed on public projects were obtained from Pakistan Manpower Institute, Islamabad. Pakistan Manpower Institute is a federal management and leadership training institute for Pakistan public sector organizations officers. We contacted project directors and explained the purpose of the research. We ensured anonymity and confidentiality. We sent them a link to an online survey thru email.

We wished them to arrange our survey data from leaders and subordinates to eradicate common source bias (Podsakoff et al., 2012). At T1, we requested them to rate TFL, TS, and JS, and give their demographics. We have given them 3 weeks. A reminder email was sent after 2 weeks. After 3 months, we asked them to assess PS. Again given them 3 weeks, a reminder email was sent after 2 weeks. With an overall response rate of 60.83%, which is satisfactory according to Pesämaa et al. (2021), we received a total of 365 finished questionnaires. Demographics are presented in Table 1.

**TABLE 1:** Demographics.

Characteristics	Category	Frequency	Cumulative percent
Gender	Male	263	80.7
	Female	63	100.0
Education	Less than 16 years	6	1.8
	16 years (BS)	220	69.3
	18 years (MS)	75	92.3
	Above 18 years (PhD)	25	100.0
Age group	25–30	89	27.3
	31–35	47	41.7
	36–40	45	55.5
	41–45	75	78.5
	46–50	25	86.2
	51–55	34	96.6
	56–60	2	97.2
	61 and above	9	100.0
Experience	1–5	139	42.6
	6–10	20	48.8
	11–15	20	54.9
	16–20	37	66.3
	21–25	84	92.0
	26–30	22	98.8
	31 and above	4	100.0
Position	Project managers	150	46.0
	Team members	176	100.0
Project type	Construction	41	12.6
	Information technology	74	35.3
	Environment	60	53.7
	Engineering	53	69.9
	Technology	47	84.4
	Education	51	100.0

Before the statistical analysis, the data were carefully scrutinized for missing values, outliers, multi-collinearity, and normality. We used the AMOS-21 and SPSS-21 software for statistical analysis. Skewness and kurtosis values were found within the acceptable range. Variance inflation factor (VIF) values for all constructs were below 3. Furthermore, we have used Harman’s single factor (HSF) to estimate the common method bias (CMB). HSF is a very effective technique to estimate CMB (Pesämaa et al., 2021). Our results confirmed that CMB is not a major concern for this research.

### Research instrument

This study involved one independent variable (TFL), two mediators (TS and JS), and one dependent variable (PS). The measures used for this study were espoused from earlier studies. All measures were validated and reliable. We used a 5-point Likert Scale (1 = Strongly Disagree to 5 = Strongly Agree) to rate variables. The measurement instruments for each of the variables are described below.

#### Trust

Gillespie’s (2003) scale was used to measure TS, and this scale has 11 items. The reliability of the scale is 0.84.

#### Job satisfaction

We used Cammann et al.’s (1983) scale to measure JS. This scale has three items. The reliability of the scale is 0.82.

#### Transformational leadership

This study used Aga et al.’s (2016) scale to measure TFL. Aga et al. (2016) adopted Bass and Avolio’s (2000) MLQ to rate TFL, as this is the most utilized scale for TFL. This scale has 13 items. The reliability of the scale is 0.85.

#### Project success

We used Musawir et al.’s (2017) scale to assess PS. The scale has three dimensions: PIS, POS, and PMS. Studies have revealed that this scale has been reliable and validated. This scale has 11 items. The reliability of the scale is 0.91.

#### Control variables

“One form of endogeneity is omitted variable bias. By including relevant (and only relevant) control variables, we come much closer to the truth” (Pesämaa et al., 2021, p. 220). Therefore, we used gender, age, education, and experience as control variables. These variables mentioned above are relevant to PS, and studies have recommended the use of these as control variables (Aga et al., 2016).

### Reliability and validity

To check the reliability of the scale, generally, internal consistency is used. Churchill (1979) approves the usage of Cronbach’s alpha (α) for appraising the quality of the scale. Anderson and Gerbing (1998) recommended 0.7 or above values of Cronbach’s alpha (α). Hair et al. (2010) recommended exploratory factor analysis (EFA) and confirmatory factor analysis (CFA) for the confirmation of construct validity. First, EFA was used to check the construct validity. Our results established that all the constructs have eigenvalues above 1, and factor loadings were greater than 0.5. As recommended by Hair et al. (2010), KMO values must be larger than 0.60, Barlett’s Test of Sphericity must be significant (*p* < 0.001), and correlation among variables must be larger than 0.30 (results are presented in Table 2).

**TABLE 2:** KMO.

Variable	KMO	Bartlett’s test of sphericity	DF	*P*-value	Cronbach’s α
TS	0.78	478.85	36	0.000	0.84
TFL	0.80	964.39	55	0.000	0.85
JS	0.75	52.63	3	0.000	0.82
PS	0.84	1120.86	55	0.000	0.91

Anderson and Gerbing’s (1998) CFA model was applied to verify the analytical data. RMSEA values in the range of 0.03–0.08, and TLI and CFI values larger than 0.90 were proven to provide a good fit for the model (Hair et al., 2010; see Table 3).

**TABLE 3:** Model fit.

CMIN/Df	TLI	GFI	CFI	RMSEA
1.96	0.95	0.96	0.96	0.03

Lastly, CR and AVE are generally used to confirm the convergent and discriminatory validities (Fornell and Larcker, 1981). AVE values should be greater than or equal to 0.50, whereas CR values should be greater than 0.60 for convergent validity (Bagozzi and Yi, 1988). The square root of a construct’s AVE must surpass the construct’s correlation with other constructs in the model to confirm discriminant validity. Our results established the discriminator and convergent validities.

## Results

### Descriptive statistics and correlation

All variables confirmed significant correlation and stable consistency. Our results (Table 4) from the statistical analysis were in the satisfactory range and were statistically significant (*p* < 0.05).

**TABLE 4:** Descriptive statistics and correlation.

Variable	Mean	*SD*	1	2	3	4	5	6	7	8
Age	1.19	0.39	1							
Gender	3.16	1.86	−0.14**	1						
Education	2.36	0.65	–0.03	0.02	1					
Experience	2.96	1.93	0.11*	–0.03	0.02	1				
TFL	3.05	0.69	0.07	–0.00	0.04	0.06	1			
TS	2.97	0.59	0.10	0.02	0.05	0.05	0.67**	1		
JS	3.17	0.78	0.07	0.07	0.01	0.13*	0.41**	0.60**	1	
PS	3.21	0.58	0.07	0.03	–0.00	0.09	0.54**	0.54**	0.50**	1

*p < 0.05, **p < 0.01, ***p < 0.001.

### Mediation analysis

**FIGURE 2:**
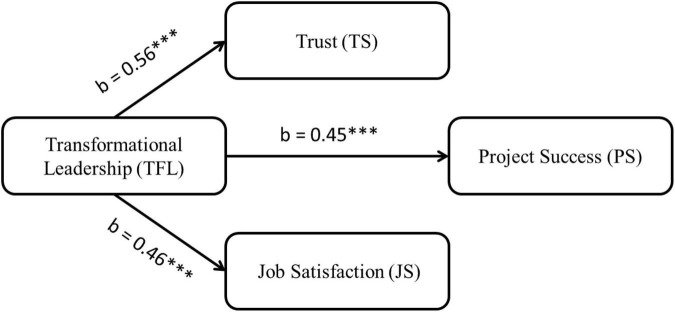
Model 1. X^2^/df = 1.94, *p* < 0.001, RMSEA = 0.035, GFI = 0.963, CFI = 0.962, TFI = 0.965. *p < 0.05, ^**^p < 0.01, ^***^p < 0.001.

We employ structural equation modeling (SEM) to test our parallel multiple mediator model (Jöreskog, 1993). We selected this method because SEM allows us to study latent variables and directly measured variables. The use of latent variables eliminates the influence of the unreliability of the mediating variable and improves the precision of the measurement of the mediating effect. Consequently, the latent variable method must have higher statistical power to detect mediating effects than the traditional regression analysis (Kline, 2011; Hair et al., 2014). We choose a parallel multiple mediation model to test our mediation hypothesis (Preacher and Hayes, 2008). We selected this method for several reasons. First, this approach minimizes the possibility of parameter bias (due to omitted variables) in multiple parallel mediators. Second, this method allows us to control multiple mediators. Third, the method controls the possible inter-correlation between mediators in a multiple mediator model. We tested our structural model in two phases. In Phase 1 (Model 1), we examine the relationship between TFL and three variables: trust, job satisfaction, and project success. All three relationships are significant and positive as shown in Figure 2. In addition, we tested the direct impact of trust and job satisfaction on project success. Both these variables had a significant positive impact on the success of the project. We used gender, age, education, and experience as control variables.

**FIGURE 3:**
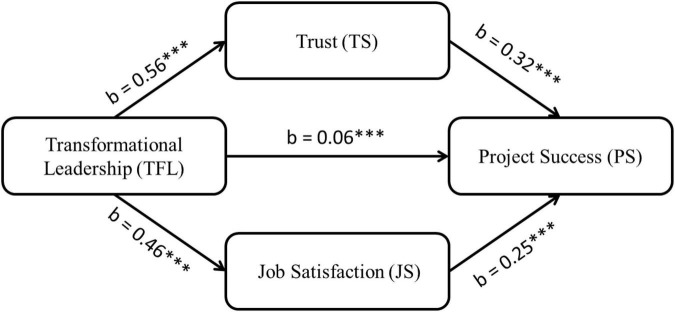
Model 2. X^2^/df = 1.93, *p* < 0.001, RMSEA = 0.031, GFI = 0.969, CFI = 0.971, TFI = 0.968. *p < 0.05, **p < 0.01, ***p < 0.001.

In Phase 2, we compared Model 1 with Model 2 (as shown in Figure 3) to identify the multiple mediating effects of trust and job satisfaction, where Model 2 includes both the link and mediator-dependent variables. At this stage, we determine if the mediation affects the success of the project when the independent variable (TFL) is controlled. If trust and job satisfaction fully mediate the relationship between TFL and project success, the path between them must become insignificant. Next, we performed supplemental tests using the bootstrap method with 5,000 samples and 95% bias-corrected confidence intervals (Efron and Tibshirani, 1994; Shrout and Bolger, 2002). Bootstrapping offers a reasonable and the most efficient method for attaining confidence limits for mediation effects under various conditions (Preacher and Hayes, 2008). While the lower and upper bounds of indirect (measured) variables do not include zero, the direct effect of TFL on project success does include zero. These results confirm our hypotheses H3 and H5.

## Discussion

The current research contributes to the literature on TFL, trust, and job satisfaction by delivering a more nuanced comprehension of the mediating role that trust and job satisfaction play in the relationships between TFL and project success.

### Hypothesis testing

H1: “Transformational leadership positively and significantly influences project success” is accepted as path coefficient is significant (b = 0.45, se = 0.07, *p* < 0.001). The PMGs’ TFL behavior is significantly correlated with project performance (Burke et al., 2006; Kissi et al., 2013; Aga et al., 2016; Naeem and Khanzada, 2016; Maqbool et al., 2017; Zaman et al., 2019). A PMG with TFL knows their employees’ needs, meets them, understands what motivates them, and endorses their welfare while accomplishing project goals (Barling et al., 2000).

H2: “Team members’ trust in project manager positively and significantly influences project success” is also accepted as path coefficient is significant (b = 0.32, se = 0.06, *p* < 0.001).

H3: “Team members’ trust in project manager has a positive mediating effect on the relationship between TFL and PS” is accepted as the indirect effect of transformational leadership on project success through the trust was significant (Indirect Effect = 0.28; 95% CI [0.24, 0.68]).

H4: “Job satisfaction positively and significantly influences project success” is accepted as path coefficient is significant (b = 0.25, se = 0.08, *p* < 0.001).

H5: “Job satisfaction has a positive mediating effect on the relationship between TFL and PS” is accepted as the indirect effect of transformational leadership on project success through job satisfaction was significant (Indirect Effect = 0.22; 95% CI [0.15, 0.55]).

### Theoretical implications

Although TFL is arguably the most researched leadership theory, empirical findings of the mediating impact of trust and job satisfaction on the association between TFL and project success have been principally missing as yet. Based on the nature of TFL and its impact on followers’ performance, we studied the relations between TFL, trust, job satisfaction, and project success, including investigations of mediating behaviors of both trust and job satisfaction. We believe that our research extends prior research on leadership in several ways. First, this research is the first study to have revealed the impact of TFL on project success through trust and job satisfaction. We provided empirical evidence that trust and job satisfaction mediated the relationship between TFL and project success. The finding underlines the close relationship between transformational leaders’ motivational and inspirational behavior and project success, while team trust and job satisfaction may impact other essential teams’ outcome variables (for example, lower levels of conflict). Second, because our findings emphasize the importance of TFL, organizations must encourage PMGs to think about the group and individual perceptions of their behavior and how TFL can be assisted at both levels. Previous studies indicate that TFL can be trained (Barling et al., 1996). Our findings propose that organizations must offer training programs that cater to TFL behaviors and give managers compulsory skills and knowledge.

Third, our results accentuate the effect of trust and job satisfaction. Managers must focus on building trust in each employee’s relationship. Furthermore, it is essential to note that TFL positively affects job satisfaction and trust among TMs. Therefore, it will be helpful to educate managers about its significant influence on TMs ‘ mutual trust and job satisfaction. Specifically, they should promote an open climate for exchange and debate with individual employees and between employees working together as a team (Eisenbeiss et al., 2008), such as through continuous team thinking. PMGs must also be mindful of the negative aspects of their impact, as employees’ perceptions of breaches of trust can have damaging effects on team performance and organizational function (Schoorman et al., 2007). Lastly, although public sector projects shape the future of our society and leadership is expected to play a significant role in these projects, research in this context is mainly sparse. Our research is the first to empirically exhibit the influence of TFL on project success with the positive mediating effects of trust and job satisfaction in the public sector.

### Managerial implications

To boost their subordinates’ job outcomes, managers must focus their attention on how their TFL behavior adds to the creation of trust and job satisfaction. They should pay attention to evolving the social sharing relationship by developing interpersonal relationships with their TMs, which will help cultivate trust. Strategies that leaders can use to improve the social sharing process may include providing individual support and encouragement to subordinates, allowing them to become further responsible for decision-making, and engaging TMs in collaborative communication (Avolio and Bass, 1995; Jung and Avolio, 2000; Dirks and Ferrin, 2002; Schaubroeck et al., 2011). Training programs that focus on the use of such approaches by PMGs at the place of work can help stimulate work outcomes from subordinates related to trust (Yang et al., 2009).

Furthermore, our findings offer significant implications for employees. It is indispensable that employees sustain a robust personal relationship (i.e., cultivate trust) with their PMG to maintain high-performance levels. It will be helpful to identify the manager’s main business and personal interests and pursue to grow common interests with them. Simultaneously, however, employees must beware of over-reliance on their PMG and be proactive in the place of work. They must be willing to present their ideas without the supervisor’s inspiration to contribute to the organization’s effectiveness. Finally, TFL should be considered when hiring, promoting, and training public PMGs. Specifically, leadership development in the public sector will benefit from implementing plans of training studies and joint training based on the concept of TFL.

### Limitations and future research

It is imperative to accentuate several limitations in the current study before spotting prospective areas for future research. First, due to the cross-sectional nature of this study, it is not easy to determine the real direction of causality between the variables studied in the research. For instance, employees who have more trust in their leader are better able to assess their leader’s TFL. To cater to this problem, future studies may implement a longitudinal study design that may help examine developmental processes for increasing followers’ trust in leaders. Second, the sample used in this study was drawn from Pakistan public sector organizations. Future work needs to be done in a bigger number of cultural and industry settings to decide the generalizability of the current study’s findings. Cross-cultural research could also be steered to enlighten the amount to which the influence of trust and job satisfaction correlates with followers’ responses to culturally transformational leaders, particularly the differences between public and private sector employees’ perceptions. It is imperative to study whether our findings are applicable to individual cultures where interpersonal relationships are less critical for organizational success.

Third, multilevel investigation can be applied in future research to cumulate followers’ assessments of TFL behavior down to the team level. Future studies may also examine potential elements that may mitigate the mediating impact of trust and job satisfaction on the association between TFL and project or organization success. These elements may include the time length the follower and leader have worked together, the employee’s personality, organizational focus, and individual cultural values (Ng and Chua, 2006). This will allow scholars to pursue an answer for whom and in what kinds of contexts or settings trust and job satisfaction will mediate the association between TFL and project success. Scholars may also deliberate how trust and job satisfaction can mediate the influence of other leadership styles on project success in future research. It may be imperative to study how trust and job satisfaction can mediate the influence of TFL on project success, for example, in manufacturing, where performance-based reward systems are widely used in an industrial setting.

## Conclusion

The volume of research on TFL has developed in the last three decades. Despite this, scholars are only just beginning to be interested in the mechanisms by which TFL transforms into follow-up action results that benefit organizations. The current research contributes to the literature by underscoring the significance of trust and job satisfaction, particularly in public sector projects, in clarifying why transformational leaders can make their subordinates demonstrate superior commitment, work harder for their companies, and engross in productive behaviors of organizational and project success. This study also augments the prior body of knowledge by underlining that transformational leaders who generate superior trust levels and satisfaction can significantly impact the performance of their followers.

## Data availability statement

The original contributions presented in this study are included in the article/supplementary material, further inquiries can be directed to the corresponding author.

## Ethics statement

Ethical review and approval was not required for the study on human participants in accordance with the local legislation and institutional requirements. Written informed consent for participation was not required for this study in accordance with the national legislation and the institutional requirements.

## Author contributions

MZF contributed to conception and design of the study, wrote the first draft of the manuscript, and performed the statistical analysis. QS supervised and reviewed the article, validated the results, and arranged funding. MA, KM, and MMF reviewed the article and validated the results. All authors contributed to manuscript revision, read, and approved the submitted version.
